# Effect of serotonin modulation on dystrophin-deficient zebrafish

**DOI:** 10.1242/bio.053363

**Published:** 2020-08-28

**Authors:** Janelle M. Spinazzola, Matthias R. Lambert, Devin E. Gibbs, James R. Conner, Georgia L. Krikorian, Prithu Pareek, Carlo Rago, Louis M. Kunkel

**Affiliations:** 1 Division of Genetics and Genomics, Boston Children's Hospital, Boston, MA, USA; 2 Department of Pediatrics, Harvard Medical School, Boston, MA, USA; 3 DMD Therapeutics Inc., Seattle, WA, USA; 4 The Stem Cell Program, Boston Children's Hospital, Boston, MA, USA; 5 Harvard Stem Cell Institute, Cambridge, MA, USA; 6 The Manton Center for Orphan Disease Research at Boston Children's Hospital, Boston, MA, USA

**Keywords:** Duchenne muscular dystrophy, Zebrafish, Serotonin, Drug screening

## Abstract

Duchenne muscular dystrophy (DMD) is a progressive muscle-wasting disease caused by mutation of the *dystrophin* gene. Pharmacological therapies that function independently of dystrophin and complement strategies aimed at dystrophin restoration could significantly improve patient outcomes. Previous observations have suggested that serotonin pathway modulation ameliorates dystrophic pathology, and re-application of serotonin modulators already used clinically would potentially hasten availability to DMD patients. In our study, we used dystrophin-deficient *sapje* and *sapje-like* zebrafish models of DMD for rapid and easy screening of several classes of serotonin pathway modulators as potential therapeutics. None of the candidate drugs tested significantly decreased the percentage of zebrafish exhibiting the dystrophic muscle phenotype in the short-term birefringence assay or lengthened the lifespan in the long-term survival assay. Although we did not identify an effective drug, we believe our data is of value to the DMD research community for future studies, and there is evidence that suggests serotonin modulation may still be a viable treatment strategy with further investigation. Given the widespread clinical use of selective serotonin reuptake inhibitors, tricyclic antidepressants and reversible inhibitors of monoamine oxidase, their reapplication to DMD is an attractive strategy in the field's pursuit to identify pharmacological therapies to complement dystrophin restoration strategies.

## Abstract (precis)

**Summary:** Phenotypic screening of serotonin pathway modulators has been performed in DMD zebrafish in order to identify small-molecules that limit the dystrophic muscle phenotype and lengthen the lifespan as potential therapeutics.

## INTRODUCTION

Duchenne muscular dystrophy (DMD) is a progressive x-linked muscle-wasting disease that affects approximately one in 4000 male births (Emery et al., 2015) in which mutations in the *dystrophin* gene result in production of a truncated, non-functional dystrophin protein (Hoffman et al., 1987; Monaco et al., 1986). Absence of dystrophin at the sarcolemma increases muscle susceptibility to contraction-induced damage (Dellorusso et al., 2001) and causes alterations in signaling pathways (Acharyya et al., 2007; Allen et al., 2016; Feron et al., 2009; Garbincius and Michele, 2015; Spinazzola et al., 2015) that lead to cycles of myofiber degeneration, regeneration, and fibrosis (Cros et al., 1989; Marshall et al., 1989). The consequent muscle weakness causes loss of independent ambulation between 10 and 12 years of age, and premature death occurs in the late twenties to early thirties typically due to cardiorespiratory failure (Emery et al., 2015).

Although glucocorticoid therapy, combined with advances in respiratory supportive care, have improved quality of life and extended life expectancy (Biggar et al., 2006; Gloss et al., 2016; Sheehan et al., 2018), there is no cure for DMD. Currently, there are several treatment strategies under investigation aimed at restoration of dystrophin expression, such as viral delivery of micro-dystrophin and read-through of translation stop codons (Verhaart and Aartsma-Rus, 2019). Notably, Eteplirsen and Golodirsen, two drugs that act to promote dystrophin production by restoring the translational reading frame of *dystrophin*, have recently been approved by the FDA (Aartsma-Rus and Corey, 2020; Frank et al., 2020; Mendell et al., 2013). However, these therapies are not expected to cure DMD given that they result in production of a low abundance of truncated, partially functional forms of dystrophin protein, and a dramatic change in the course of the disease will likely require a combinatorial treatment approach (Verhaart and Aartsma-Rus, 2019). Thus, identification of therapies that improve pathology independent of dystrophin and work complementarily with genetic-based approaches would be of significant value to patients.

Interestingly, there are several previous studies suggesting serotonin modulation may be a candidate strategy to treat muscular dystrophy. Serotonin is a neurotransmitter most commonly associated with the regulation of homeostatic processes including sleep, appetite, emotions and perception (Mohammad-Zadeh et al., 2008). Thus, serotonin, its precursors and products, and serotonin modulators such as selective serotonin reuptake inhibitors (SSRIs), tricyclic antidepressants and reversible inhibitors of monoamine oxidase (RIMAs) are commonly prescribed clinically to treat insomnia, depression and anxiety (Taciak et al., 2018). However, even prior to the discovery of the *dystrophin* gene, treatment of dystrophic chickens with the serotonin antagonist methysergide was found to prevent muscle weakness and reduce serum creatine kinase (Bhargava et al., 1977; Hudecki and Barnard, 1976). More recently, investigation of serotonin modulators have been investigated in *C. elegans*, mouse and zebrafish models of DMD. In a *C. elegans* model of DMD, treatment with serotonin or the SSRIs fluoxetine, imipramine or trimipramine suppressed muscle degeneration, and reduction of serotonin levels caused degeneration of non-dystrophic muscles (Carre-Pierrat et al., 2006). *Mdx* mice treated with the tricyclic antidepressant amitriptyline exhibited decreased forelimb muscle pro-inflammatory cytokines TNF-α and IL-6 (Manning et al., 2014), and Gurel et al. found that serotonin, in combination with histamine, improved grip strength and lowered contraction-induced injury in *mdx^5cv^* mice (Gurel et al., 2015). In dystrophin-deficient *sapje* zebrafish, fluoxetine was found to prevent muscle pathology and disruption of muscle membrane integrity, and transcriptome analysis indicated changes in calcium homeostasis as a potential mechanism of extracellular serotonin-induced rescue of dystrophin deficiency (Waugh et al., 2014).

Zebrafish have emerged as a powerful preclinical genetic model to study muscle development and diseases, complement murine studies, and accelerate the discovery of potential therapeutics. The zebrafish dystrophin associated protein complex (DAPC) localizes to the muscle cell membrane and functions similarly as in mammals (Guyon et al., 2003). The highly ordered sarcomeric structure of zebrafish somatic muscle can be observed as bright chevrons on a dark background by rotating polarized light through the transparent zebrafish embryo. This optical property, known as birefringence, results from the diffraction of polarized light through the pseudo-crystallin array of muscle sarcomeres, and can thus be used as an assay to detect the disorganized muscle structure characteristic of diseased muscle repeatedly and noninvasively. The two DMD zebrafish lines, *sapje* and *sapje-like*, harbor mutations in the *dystrophin* gene that both result in absence of the dystrophin muscle protein causing extensive muscle degeneration, inflammation, and fibrosis similar to the pathogenesis of human DMD (Bassett and Currie, 2004; Guyon et al., 2009). Mutant fish exhibit a patchy birefringence pattern detectable 4 days post fertilization (dpf) and death occurs prematurely, typically beginning around 12 dpf.

In this study, we used *sapje* and *sapje-like* zebrafish to assess serotonin and 16 serotonin precursors, products and modulating drugs as DMD therapeutics. We performed both short-term birefringence assays to assess the ability of the candidate drugs to prevent manifestation of the dystrophic phenotype as well as long-term survival assays. Unfortunately, our experiments did not recapitulate previous positive results, but should be taken into account in future efforts to assess serotonin modulation as a strategy for ongoing DMD therapy development.

## RESULTS

### Short-term drug screening in *sapje* and *sapje-like* zebrafish by birefringence assay

**Fig. 1.**
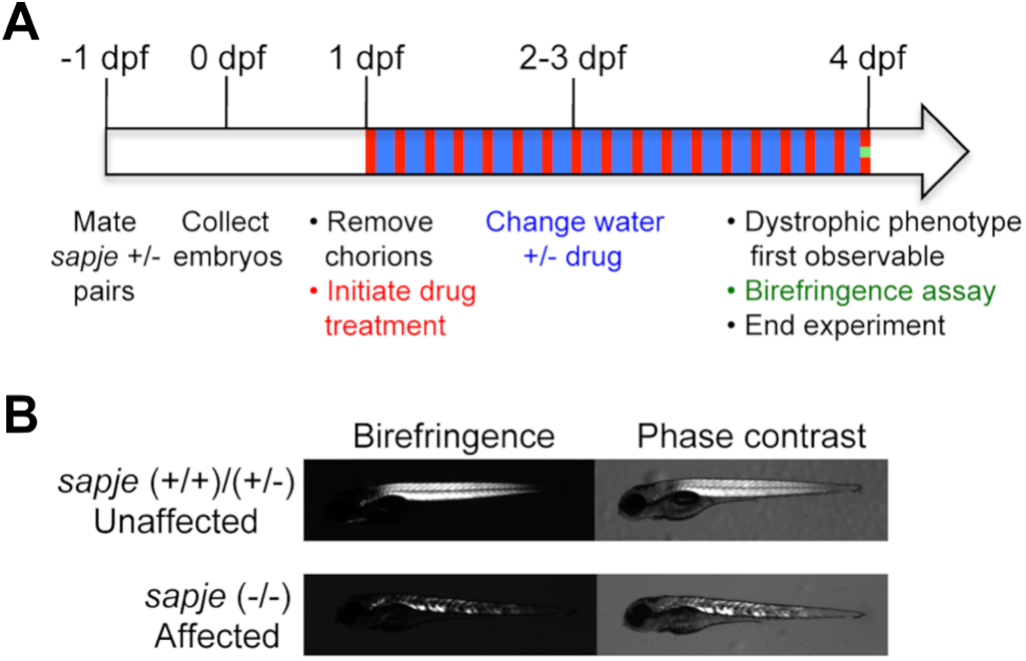
**Experimental design of the short-term zebrafish birefringence assay.** (A) Heterozygous *sapje* or *sapje-like* pairs were mated and their respective embryos were collected and pooled. Drug treatment was initiated on 1 dpf and continued through 4 dpf when birefringence was analyzed. (B) Representative images of the patchy muscle birefringence pattern characteristic of *sapje* and *sapje-like* homozygous mutants compared to the highly organized sarcomere structure of (+/+) and (+/−) siblings. Given that the *sapje* and *sapje-like* dystrophin mutations are recessive, 25% of untreated offspring are expected to exhibit the affected muscle phenotype.

The short-term assay (Fig. 1A) assessed the efficacy of our candidate drugs (Table 1) to prevent manifestation of the *sapje*/*sapje-like* homozygous mutant muscle phenotype detected by birefringence assay (Fig. 1B). In short, 1 dpf embryos resulting from heterozygous pair matings were treated either a candidate drug, 0.1% dimethyl sulfoxide (DMSO) control, or E2 water (untreated). On 4 dpf, fish were analyzed by birefringence assay in which polarized light is passed through the transparent zebrafish body to detect either the highly ordered ‘unaffected’ sarcomeric structure of normal zebrafish somatic muscle or the patchy ‘affected’ phenotype characteristic of homozygous mutant *sapje* and *sapje-like* fish. Because the *sapje* and *sapje-like* dystrophin mutations are recessive, approximately 25% of embryos from mating heterozygous pairs are expected to exhibit the affected birefringence muscle phenotype. Thus, we used this value as a basis for our DMSO and untreated control groups, and assessed whether each drug significantly decreased this percentage. We used the non-selective phosphodiesterase (PDE) inhibitor aminophylline as our positive control, which was discovered as a positive effector in a previous zebrafish drug screen in our lab and also confirmed independently (Hightower et al., 2020; Kawahara et al., 2011; Waugh et al., 2014). Aminophylline (2.5 μg/ml) consistently decreased the percentage of affected fish to 10–15% in our experiments.

**Table 1.**
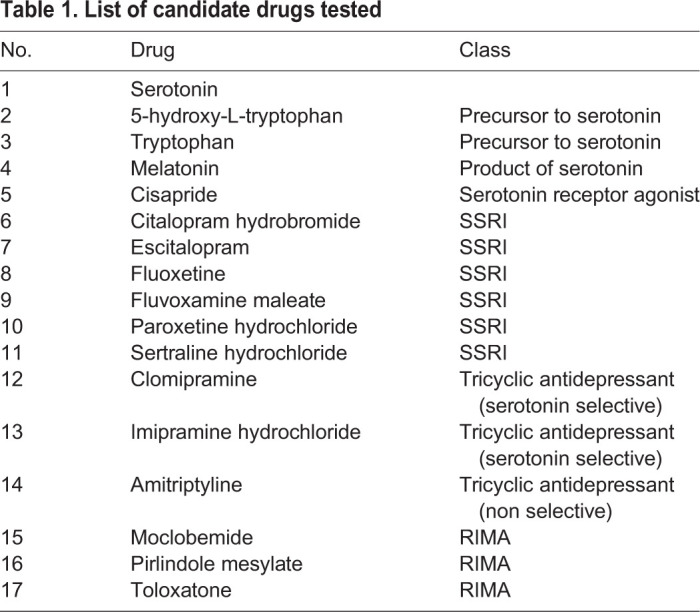
**List of candidate drugs tested**

### Serotonin, serotonin precursors, products and receptor agonists

**Fig. 2.**
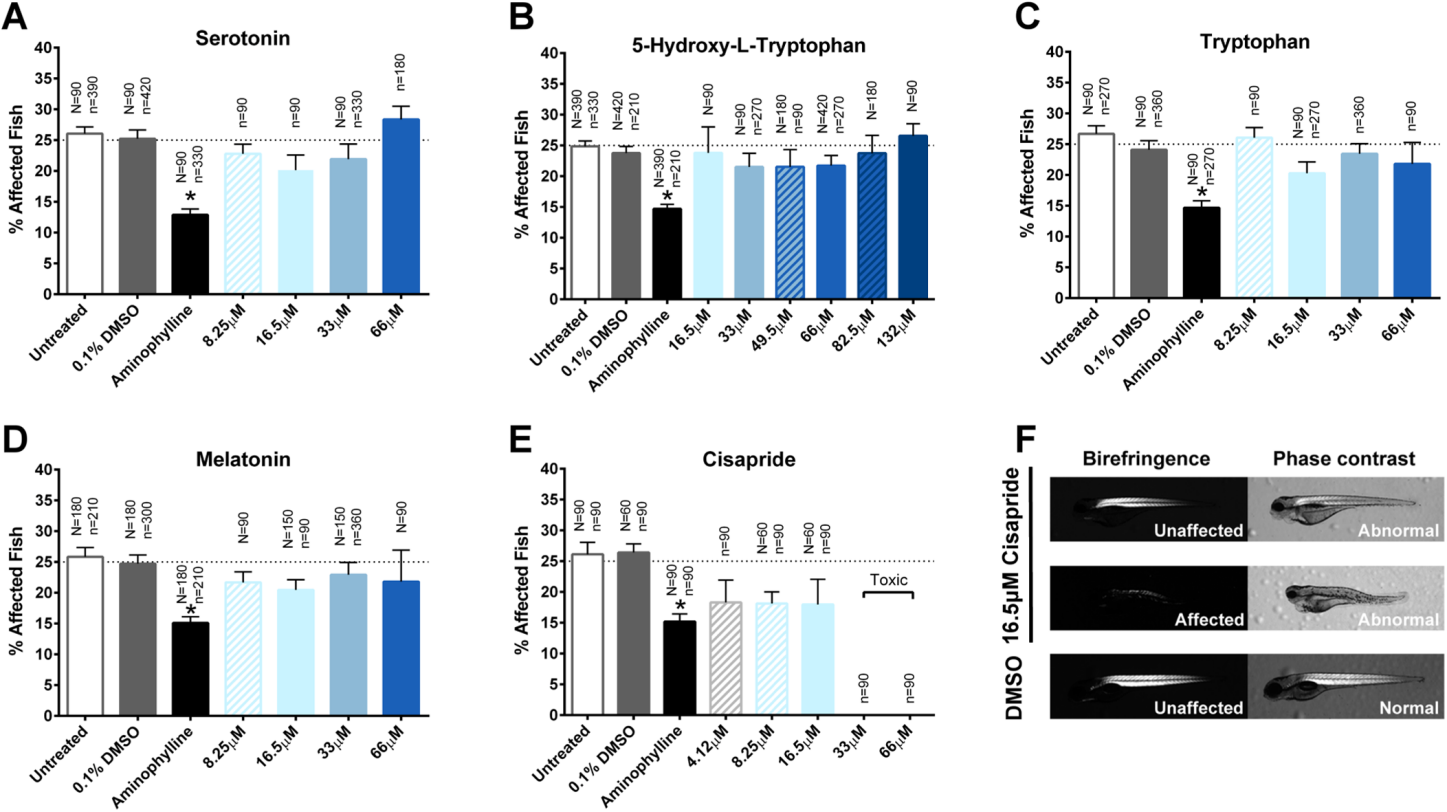
**Short-term assay of serotonin, serotonin precursors, products and receptor agonists.** (A–E) Treatment with serotonin, 5-hydroxy-L-tryptophan (5-HTP), tryptophan, melatonin and cisapride did not significantly decrease the percentage of zebrafish exhibiting the affected muscle phenotype detected by birefringence. Treatment with 2.5 μg/ml aminophylline significantly decreased the percentage of affected fish. Data represent means±s.e.m.; *P<0.05 versus paired control by one-way ANOVA and Bonferroni post-hoc test. Values above each column indicate the number of *sapje* (*N*) and *sapje-like* (*n*) fish treated with the respective drug. (F) Both affected and unaffected zebrafish treated with ≤16.5 μM cisapride exhibited abnormal body morphology.

Our first set of candidate drugs included serotonin, the serotonin precursors 5-hydroxy-L-tryptophan (5-HTP) and tryptophan, the serotonin product melatonin and the serotonin receptor agonist cisapride. Short-term treatment with serotonin (8.25 μM–66 μM), 5-HTP (16.5 μM–132 μM), tryptophan (8.25 μM–66 μM), melatonin (8.25 μM–66 μM), and cisapride (4.12 μM–66 μM) did not significantly decrease the percentage of affected fish (Fig. 2A–E). Cisapride was toxic at doses of 33 μM and above and caused morphological abnormalities in both affected and unaffected fish at non-toxic doses (Fig. 2E,F).

### SSRIs

**Fig. 3.**
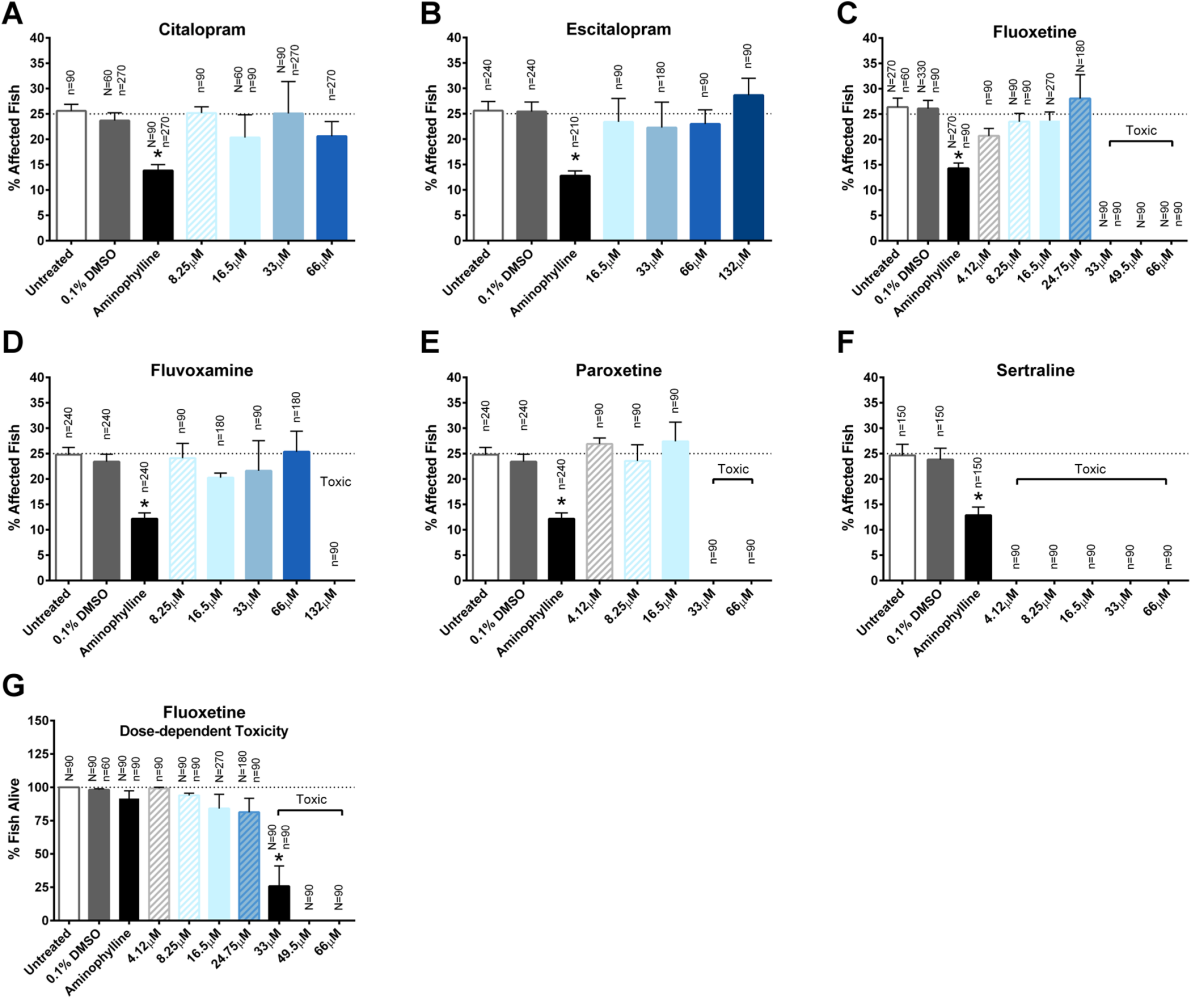
**Short-term assay of SSRIs.** (A–F) Treatment with citalopram, escitalopram, fluoxetine, fluvoxamine, paroxetine and sertraline did not significantly decrease the percentage of zebrafish exhibiting the affected muscle phenotype detected by birefringence. Treatment with 2.5 μg/ml aminophylline significantly decreased the percentage of affected fish. (G) Zebrafish treated with fluoxetine exhibited dose-dependent toxicity. Data represent means±s.e.m.; *P<0.05 versus paired control by one-way ANOVA and Bonferroni post-hoc test. Values above each column indicate the number of *sapje* (*N*) and *sapje-like* (*n*) fish treated with the respective drug.

Our second set of candidate drugs included the SSRIs citalopram, escitalopram, fluoxetine, fluvoxamine, paroxetine and sertraline. Short-term treatment with citalopram (8.25 μM–66 μM), escitalopram (16.5 μM–132 μM), fluoxetine (4.12 μM–66 μM), fluvoxamine (8.25 μM–132 μM), paroxetine (4.12 μM–66 μM) and sertraline (4.12 μM–66 μM) did not significantly decrease the percentage of affected fish (Fig. 3A–F). Fluoxetine was toxic at 33 μM and above, fluvoxamine was toxic at 132 μM, paroxetine was toxic at 33 μM and above, and sertraline was toxic at all doses tested. Fluoxetine toxicity at the 33 μM dose was particularly unexpected because it was previously found to significantly decrease the percentage of affected *sapje* fish (Waugh et al., 2014). In our experiments, fluoxetine elicited dose-dependent toxicity and was ineffective at non-toxic doses (Fig. 3C,G).

### RIMAs

**Fig. 4.**
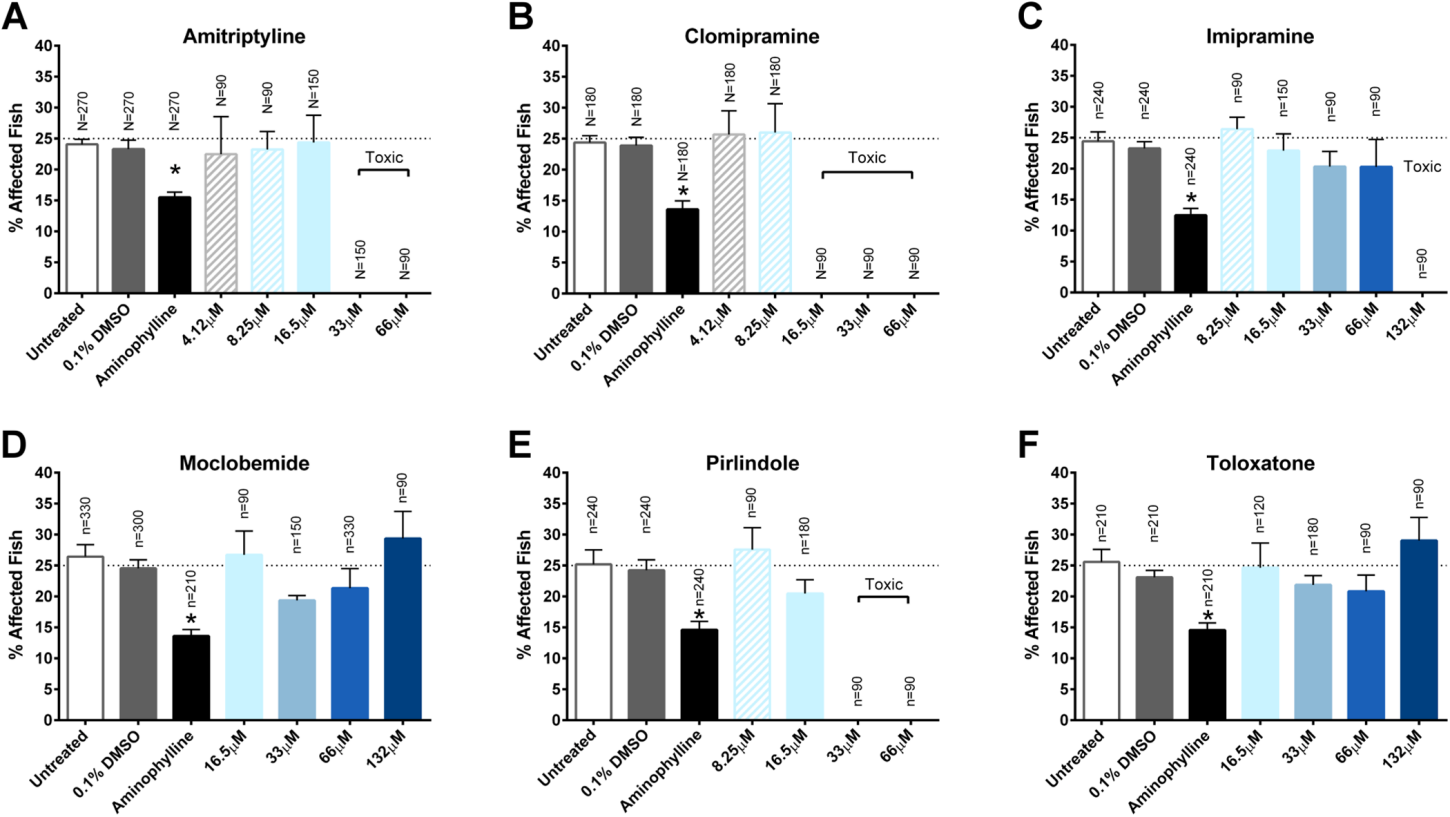
**Short-term assay of tricyclic antidepressants and RIMAs.** (A–F) Treatment with amitriptyline, clomipramine, imipramine, moclobemide, pirlindole and toloxatone did not significantly decrease the percentage of zebrafish exhibiting the affected muscle phenotype detected by birefringence. Treatment with 2.5 μg/ml aminophylline significantly decreased the percentage of affected fish. Data represent means±s.e.m.; *P<0.05 versus paired control by one-way ANOVA and Bonferroni post-hoc test. Values above each column indicate the number of *sapje* (*N*) and *sapje-like* (*n*) fish treated with the respective drug.

Our third set of candidate drugs included the tricyclic antidepressants amitriptyline, clomipramine and imipramine, and the RIMAs moclobemide, pirindole, and toloxatone. Short-term treatment with amitriptyline (4.12 μM–66 μM), clomipramine (4.12 μM–66 μM), imipramine (8.25 μM–132 μM), moclobemide (16.5 μM–132 μM), pirlindole (8.25 μM–66 μM) and toloxatone (16.5 μM–132 μM) did not significantly decrease the percentage of affected fish (Fig. 4A–F). Amitriptylline was toxic at 33 μM and above, clomipramine was toxic at 16.5 μM and above, imipramine was toxic at 132 μM and pirlindole was toxic at 33 μM and above.

### Serotonin modulators do not increase zebrafish long-term survival

**Fig. 5.**
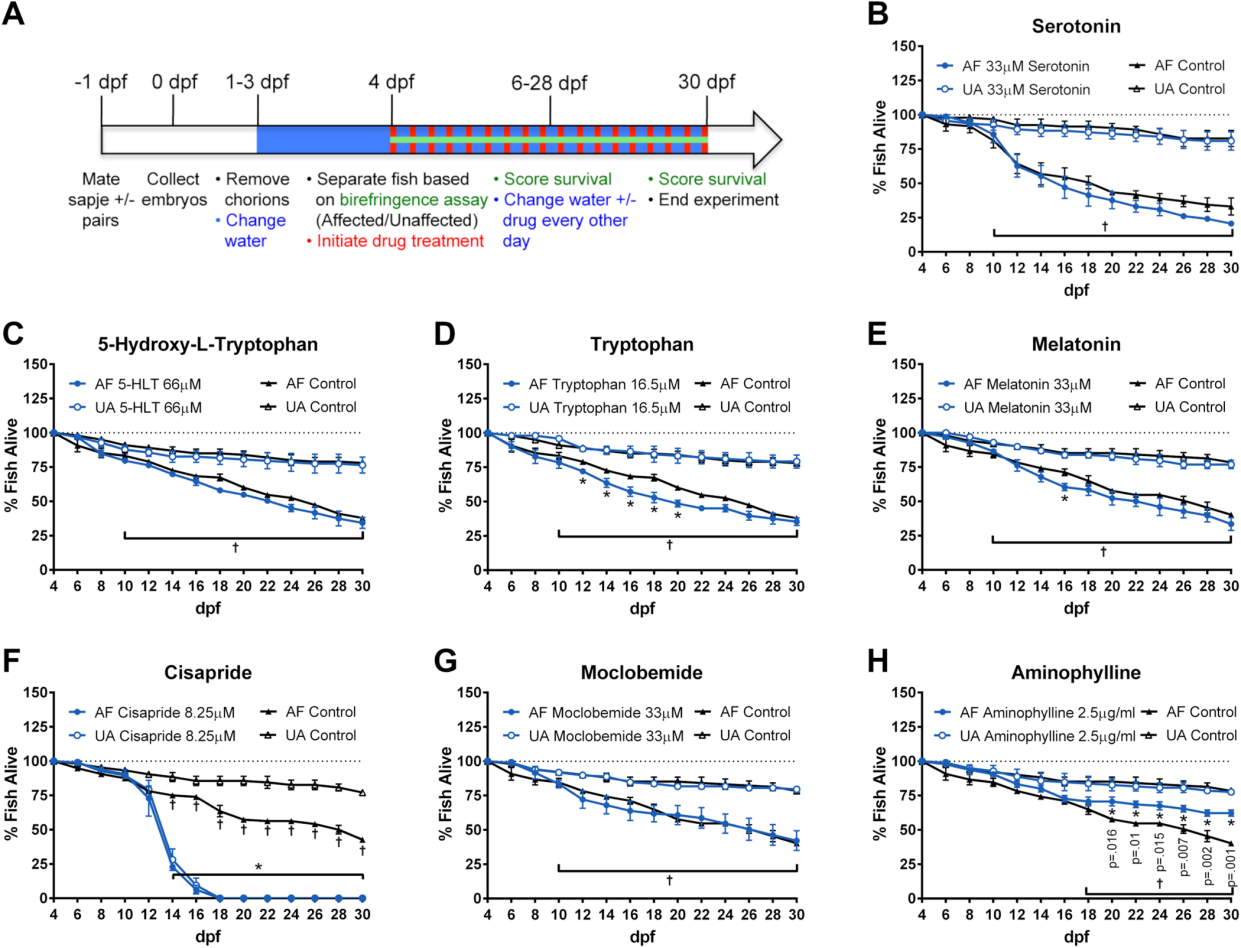
**Long-term zebrafish survival assay.** (A) Experimental design of the long-term survival assay. Cohorts of *sapje* or *sapje-like* offspring were screened as affected or unaffected on 4 dpf, at which time drug treatment was initiated and continued through 30 dpf. The water was changed and surviving fish were counted every other day. (B–G) Treatment with 33 μM serotonin, 66 μM 5-HTP, 16.5 μM tryptophan, 33 μM melatonin, 8.25 μM cisapride or 33 μM moclobemine did not significantly improve the survival of affected fish. 8.25 μM cisapride was toxic to both affected and unaffected fish beginning on 14 dpf. For each condition, 30–40 fish were tested in three replicate experiments. Data represent means±s.e.m. †*P*<0.05 affected versus respective unaffected, *P<0.05 drug-treated versus respective control by two-way ANOVA and Bonferroni post-hoc test. AF, affected; UA, unaffected. (H) Affected fish treated with 2.5 μg/ml aminophylline significantly increased survival versus affected controls. †*P*<0.05 affected versus respective unaffected, *P-values are for the closed blue circles and indicate significant difference between drug-treated AF versus control AF by two-way ANOVA and Bonferroni post-hoc test.

Several compounds that showed initial promise in the short-term assay were tested in the long-term assay to determine if they could prolong the survival of affected *sapje* fish already exhibiting the muscle phenotype. In the long-term assay, affected and unaffected fish are identified and separated by birefringence assay on 4 dpf. Drug treatment is then initiated, and the number of surviving fish in each cohort is counted every other day through 30 dpf (Fig. 5A). Affected and unaffected fish treated with 33 μM serotonin, 66 μM 5-HTP, 33 μM melatonin and 33 μM moclobemide did not exhibit increased survival compared to vehicle controls. Affected fish treated with 16.5 μM tryptophan showed significantly decreased survival from 14–20 dpf compared to affected vehicle controls. 8.25 μM cisapride was toxic to both affected and unaffected fish beginning on 14 dpf. Affected fish treated with 2.5 μg/ml aminophylline had significantly greater survival than control affected fish beginning on 20 dpf, which was consistent with previous findings (Kawahara et al., 2011).

## DISCUSSION

DMD is a multifaceted disease that will likely require a multifaceted treatment approach to address the many features of its pathology. Pharmacological therapies other than glucocorticoids to complement advancing genetic-based strategies are an emerging area of interest to improve patient outcomes (Verhaart and Aartsma-Rus, 2019). In this study, we investigated modulators of the serotonin pathway as potential candidates to treat DMD using zebrafish models of the disease. We used both a short-term birefringence assay and a long-term survival assay to assess the efficacy of several classes of serotonin pathway modulators to prevent manifestation of the dystrophic zebrafish muscle phenotype and prolong survival, respectively.

We were initially drawn to the serotonin pathway due to benefits elicited by modulators in *C. elegans*, chicken, mice and zebrafish models of DMD as previously mentioned. In particular, we were intrigued by the results of Waugh et al. who identified the SSRI fluoxetine dosed at 33 μM prevented manifestation of the *sapje* zebrafish muscle phenotype (Waugh et al., 2014). The goal of our initial experiments was to establish 33 μM fluoxetine as a serotonin-pathway positive control in addition to our standard positive control aminophylline. However, we found this dose to be toxic to both *sapje* and *sapje-like* fish and that lower doses of fluoxetine were non-toxic, but ineffective. This was observed with multiple lots of fluoxetine, which was prepared fresh for each use. It is possible that differences between studies could have been due to subtle variances in the fluoxetine stocks, which came from different sources. In addition, the independent experimental parameters differed slightly; we used 30 fish per well in six-well plates versus 20 fish per well in 24-well plates, though it seems unlikely this would have significantly impacted the results. Regardless, our disparate results with fluoxetine in the short-term *sapje* zebrafish assay highlight the importance of independent lab validation of not only serotonin modulators, but any future drugs under investigation for DMD treatment. Another consideration is that independent populations of zebrafish housed in separate facilities may develop variances over time that lead to differential responses, a factor that should be acknowledged and has not been investigated in depth to our knowledge.

As with fluoxetine, we did not observe significant positive results with any of the other candidate drugs in either short-term or long-term assays. A subset of drugs did show efficacy in preliminary experiments, which led us to test them in the long-term assay; however, additional experimental replicates indicated that they were not in fact significant. These negative results were consistent with Gurel et al. who reported that serotonin alone did not significantly improve *mdx*-mouse muscle strength, though it was effective when administered in combination with histamine (Gurel et al., 2015). Additionally, Carre-Pierrat et al. did not observe significant improvement with the 21 modulators of monoamines they tested in *mdx^5cv^* mice, though they did find that amitriptyline and imipramine modestly improved some aspects of motor function and force generation (Carre-Pierrat et al., 2011). Interestingly, studies have also reported negative effects of increased serotonin on muscle including increased serum creatine kinase, a characteristic biomarker of DMD, associated with serotonin modulating antipsychotic drugs (Meltzer, 2000). In fact, serotonin has been used to induce myopathy in rats to model dystrophic muscle degeneration and regeneration (Narukami et al., 1991).

Despite our negative results, there is genetic evidence suggesting that serotonin modulation may still be a viable DMD therapeutic strategy. *Morpholino* gene knockdown of slc6a4, the serotonin transporter, has been shown to prevent phenotype development in *sapje* zebrafish (Waugh et al., 2014). The mechanism by which serotonin modulators improved dystrophic pathology in previous studies is unknown, and it is possible that they were functioning to modulate blood flow, as serotonin has been shown to regulate vascular tone (Côté et al., 2004). DMD patients have been shown to have lower levels of serotonin uptake in platelets (Arora et al., 1987; Murphy et al., 1973), which mediate vascular homeostasis and may influence DMD ischemia. Serotonin has also been implicated in insulin secretion and glucose uptake (Hajduch et al., 1999), and may interact with myostatin to regulate glucose metabolism in skeletal muscle (Chandran et al., 2012). Use of SSRIs is known to affect muscle function and energy metabolism in skeletal muscle tissue (Visco et al., 2018). Hence, further investigation of the mechanisms by which serotonin modulation impacts muscle health may guide research towards an effective pharmacologic treatment, perhaps by means other than our candidate drugs.

Although we did not observe positive results with the serotonin modulators, we did observe significant efficacy with 2.5 μg/ml aminophylline, a non-specific PDE inhibitor, in both the short- and long-term zebrafish assays. This is consistent with and reaffirms the results of Kawahara et al. (2011), who first identified aminophylline to ameliorate the dystrophic phenotype of *sapje* zebrafish, as well as the results of subsequent investigators (Hightower et al., 2020; Waugh et al., 2014). Despite variable clinical trial success with PDE5 inhibitors such as sildenafil and tadalafil and side effects associated with other non-specific PDE inhibitors such as pentoxiphylline (Spinazzola and Kunkel, 2016), our results suggest that PDEs may still be a relevant target for DMD therapeutics.

The widespread clinical use of SSRIs, tricyclic antidepressants and RIMAs made their potential reapplication to DMD an attractive therapeutic strategy to investigate. SSRIs are currently used by many DMD patients to treat depression and other psychosocial conditions (Wagner et al., 2007), but investigation of their potential benefits to the muscle disease has not been performed. In this study, we used dystrophin-deficient zebrafish to screen several of these serotonin modulators. Although our results were not positive, we believe these data are valuable to the DMD research community for future studies. Our list of candidate drugs tested in this project was not all inclusive, and there exist several other clinically utilized serotonin modulators that could be tested as potential DMD therapeutics. Furthermore, investigation of serotonin pathway modulators at lower concentrations should also be investigated, since recent study showed that application of 0.1–1 µM SSRI sertraline or escitalopram was not toxic and improved survival in a zebrafish model of MEGF10 myopathy (Saha et al., 2019); therefore, we do not exclude beneficial effect of serotonin modulation in DMD or other muscular disorders. To this end, identification of pharmacological therapies to treat the secondary consequences of dystrophin deficiency, especially via reapplication of drugs already used clinically, is an area worthy of continued investigation.

## MATERIALS AND METHODS

### Ethics statement

Zebrafish (*Danio rerio*) used in this study were handled in accordance with the Guide for the Care of Laboratory Animals of the National Institutes of Health. Humane endpoints were used during all zebrafish experiments. The specific criteria used were whether zebrafish exhibited a swim response to touch. Those that did not were euthanized with the technique appropriate for the given larval stage in accordance with the National Institute of Health Final Report to OLAW on Euthanasia of Zebrafish. Zebrafish that survived through the course of the survival study (30 dpf) were also euthanized appropriately in accordance to the National Institutes of Health Final Report to OLAW on Euthanasia of Zebrafish. Specifically, zebrafish were immobilized by submersion in ice water (five parts ice to one part water, 0–4°C) for at least 10 min following cessation of opercular (i.e. gill) movement. The protocol used in this study was approved by the Institutional Animal Care and Use Committee (IACUC) at Boston Children's Hospital (Protocol number: 18-08-3749R).

### Zebrafish husbandry and genotyping

Zebrafish were housed in the Boston Children's Hospital Aquatics Facility and maintained in accordance to IACUC standards (environmental and housing conditions are available at dx.doi.org/10.17504/protocols.io.bb2iiqce). Fertilized eggs were collected and raised in E2 water at 28.5°C (Nusslein-Volhard and Dahm, 2002). Genomic DNA was extracted and used as the PCR template. The following primer sets were used for genotyping the specific mutations in the *dystrophin* gene of *sapje* fish: forward primer 5′-CTGGTTACATTCTGAGAGACTTTC-3′; reverse primer 5′-AGCCAGCTGAACCAATTAACTCAC-3′) and *sapje-like* fish: forward primer 5′-TCTGAGTCAGCTGACCACAGCC-3′; reverse primer 5′-ATGTGCCTGACATCAACATGTGG-3′. Sequencing was preformed by the Molecular Genetics Core Facility at Children's Hospital Boston and analyzed using Sequencher.

### Short-term zebrafish assay

Embryos from heterozygous *sapje* or *sapje-like* matings were pooled and dechorionated on 1 dpf. Embryos were placed in individual wells of six-well plates with 30 embryos/well. Each well contained an experimental drug, positive control 2.5 μg/ml aminophylline, control 0.1% DMSO, or control E2 water. On 4 dpf, the dystrophic muscle phenotype was detected by using a birefringence assay as described below to discern affected versus unaffected fish.

### Birefringence assay

The *sapje*/*sapje-like* dystrophic muscle phenotype was detected by using a birefringence assay, a technique used to analyze myofiber integrity using polarized light performed as described previously (Granato et al., 1996). Polarizing filters were placed on a bottom-lit dissection scope, and images were acquired with a QImaging Retiga 2000R camera fitted to a Nikon SMZ1500 microscope using OpenLab software. Zebrafish were anesthetized with tricaine and positioned relative to the polarized light to produce maximal birefringence illumination.

### Long-term zebrafish assay

Pairs of heterozygous *sapje* or *sapje-like* fish were mated, and fertilized eggs were maintained at 28.5°C. Zebrafish embryos were pooled and dechorionated on 1 dpf and raised according to standard procedures and criteria. For long-term treatment of dystrophin-deficient fish, cohorts of fish were screened on 4 dpf by birefringence assay to identify mutant fish exhibiting the abnormal muscle phenotype and divided into affected and unaffected groups. Groups of 30–40 fish were then treated from 4 to 30 dpf in 50 ml of E2 water containing a candidate compound or vehicle control. The number of surviving fish was counted and the water changed every other day.

### Candidate drugs

The candidate drugs used were as follows: serotonin (Sigma-Aldrich), 5-hydroxy -L-tryptophan (Sigma-Aldrich), tryptophan (Sigma-Aldrich), melatonin (Sigma-Aldrich), cisapride (Sigma-Aldrich), citalopram (Sigma-Aldrich), escitalopram (Sigma-Aldrich), fluoxetine (Sigma-Aldrich), fluvoxamine (Selleckchem), paroxetine (Sigma-Aldrich), sertraline (Sigma-Aldrich), clomipramine (Sigma-Aldrich), imipramine (Sigma-Aldrich), amitriptyline (Sigma-Aldrich), moclobemide (Sigma-Aldrich), pirlindole (Santa Cruz Biotechnology), toloxatone (Sigma-Aldrich) and aminophylline (Sigma-Aldrich) (Table 1). Each candidate compound was dissolved in 0.1% DMSO and tested at the initial doses of 16.5 μM, 33 μM, and 66 μM, which were then expanded upon based on efficacy and toxicity to doses ranging from 4.12 μM–132 μM. Doses that elicited greater than 50% mortality were considered toxic and each dose was tested a minimum of three times and up to 16 times.

### Statistical analysis

All results are shown as means± standard error of the mean (s.e.m.). Statistical analyses of the data were performed using StatPlus to implement one- and two-way ANOVA followed by Bonferroni post-hoc tests. *P*-values of <0.05 were considered to be statistically significant.
